# Editorial: Reviews in experimental pharmacology and drug discovery 2023: pharmacological management of non-communicable diseases

**DOI:** 10.3389/fphar.2025.1435638

**Published:** 2025-04-01

**Authors:** Ali Sharif, Virginia Flores-Morales, Hanan Farouk Aly

**Affiliations:** ^1^ Department of Pharmacology, Institute of Pharmacy, Faculty of Pharmaceutical and Allied Health Sciences, Lahore College for Women University, Lahore, Pakistan; ^2^ Laboratorio de Síntesis Asimétrica y Bio-Quimioinformática (LSAyB), Ingeniería Química (UACQ), Universidad Autónoma de Zacatecas, Zacatecas, Mexico; ^3^ National Research Centre (Egypt), Cairo, Egypt

**Keywords:** experimental pharmacology, experimental pharmacology and drug discovery, adrenergy agonist, chemotherapy, hyperactive syndrome

Experimental pharmacology is the basis of drug discovery. The discovery of novel drugs can be done through the identification of active ingredients in traditional medicines or via searching chemical libraries for specific molecules with predicted kinetic properties. The aim of the Research Topic was to throw light on recent pharmacological advancements in managing non-communicable diseases.

The Research Topic provides insight into the evaluation of mirabegron, which is a β3-adrenergic agonist used as a medical expulsive therapy for ureteral stones in adults. One study concluded with the opinion that mirabegron appeared to a better treatment option than a placebo for stones ≤4–6 mm, especially distal ureteral stones.

Catheter-related bladder discomfort (CRBD) secondary to the use of large-diameter urinary catheters, particularly after urological surgeries, can lead to increased frequency in urination, sometimes accompanied by involuntary loss of urine. Nefopam, a centrally acting analgesic with anticholinergic properties, was evaluated for management of postoperative CRBD. Nefopam administration mitigated the frequency and severity of early postoperative CRBD without causing evident side effects.

Ondansetron was validated for its use in pregnancy-associated morning sickness, but the study lacked clarity in the identification and analysis of efficacy and safety. Similarly, ondansetron has been a gold standard in treating chemotherapy-induced nausea and vomiting. However, there is still room to improve safety and the quality of patient’s lives.

Amphetamine and its derivatives have been employed in the treatment of attention-deficit hyperactive syndrome for a long time. A study emphasized the importance of recognizing potential side effects and addiction risks for this drug.

Tyrosine kinase inhibitors are one of the mainstays in cancer therapy and were proposed to have glycemic control properties. The results of a study showed a mixture of improved glycemic control with some tyrosine kinase inhibitors but clinically insignificant hyperglycemia control with other tyrosine kinase inhibitors. The repurposing strategies of these inhibitors towards managing diabetes mellitus needs further exploration.

